# Helical Chirality: a Link between Local Interactions and Global Topology in DNA

**DOI:** 10.1371/journal.pone.0009326

**Published:** 2010-02-19

**Authors:** Youri Timsit, Péter Várnai

**Affiliations:** 1 Information Génomique et Structurale, CNRS - UPR2589, Institut de Microbiologie de la Méditerranée, Parc Scientifique de Luminy, Marseille, France; 2 Department of Chemistry and Biochemistry, School of Life Sciences, University of Sussex, Brighton, United Kingdom; Universität Heidelberg, Germany

## Abstract

DNA supercoiling plays a major role in many cellular functions. The global DNA conformation is however intimately linked to local DNA-DNA interactions influencing both the physical properties and the biological functions of the supercoiled molecule. Juxtaposition of DNA double helices in ubiquitous crossover arrangements participates in multiple functions such as recombination, gene regulation and DNA packaging. However, little is currently known about how the structure and stability of direct DNA-DNA interactions influence the topological state of DNA. Here, a crystallographic analysis shows that due to the intrinsic helical chirality of DNA, crossovers of opposite handedness exhibit markedly different geometries. While right-handed crossovers are self-fitted by sequence-specific groove-backbone interaction and bridging Mg^2+^ sites, left-handed crossovers are juxtaposed by groove-groove interaction. Our previous calculations have shown that the different geometries result in differential stabilisation in solution, in the presence of divalent cations. The present study reveals that the various topological states of the cell are associated with different inter-segmental interactions. While the unstable left-handed crossovers are exclusively formed in negatively supercoiled DNA, stable right-handed crossovers constitute the local signature of an unusual topological state in the cell, such as the positively supercoiled or relaxed DNA. These findings not only provide a simple mechanism for locally sensing the DNA topology but also lead to the prediction that, due to their different tertiary intra-molecular interactions, supercoiled molecules of opposite signs must display markedly different physical properties. Sticky inter-segmental interactions in positively supercoiled or relaxed DNA are expected to greatly slow down the slithering dynamics of DNA. We therefore suggest that the intrinsic helical chirality of DNA may have oriented the early evolutionary choices for DNA topology.

## Introduction

The topology of DNA is finely tuned by topoisomerases [1] and plays a major role in many cellular processes in both prokaryotes and eukaryotes, such as remote gene regulation and site-specific recombination [2], [3]. Maintaining the appropriate sign and density of DNA supercoiling is vital for the cell and has represented a constant evolutionary challenge [4]. Although genomic DNA is mainly negatively supercoiled ((−)sc) in mesophilic cells, transcription and DNA replication may generate domains of positively supercoiled ((+)sc) DNA *in vivo* [5]–[7]. However, (+)sc is generally considered undesirable that must be removed by gyrase or topoisomerase IV [8]. Nevertheless, a role for (+)sc has recently been suggested for the control of telomere resolution [9]. In contrast to mesophilic cells, hyperthermophilic archaea and bacteria possess a reverse gyrase that introduces permanent positive supercoiling in their genome which is thought to be required to accommodate life at elevated temperatures [4], [10], [11].

It has been proposed that factors that affect the local properties of DNA will directly influence the global properties of supercoiled DNA and, in turn, changes in superhelicity will have repercussions on the local DNA structure and stability [12]. Thus the interplay of local and global properties must be considered as a key element in the cellular function of DNA. For example, the formation of triplex or cruciform structures in specific sequences modulates the rate of encounter and the efficiency of communication between remote sites and may affect transcription through altered global dynamics of supercoiled DNA [13], [14]. Consequently, local intra- or intermolecular DNA-DNA interactions play a central role by establishing a link between the two hierarchical levels of structural organisation in DNA.

Juxtaposition of DNA double helices in a crossover arrangement represents a ubiquitous motif in higher-order DNA structures and is known to be implicated in genetic functions such as recombination, gene regulation and chromatin packaging [2], [15]. Moreover, it has been found that DNA crossovers are in fact the substrates to topoisomerases II [16]. Interestingly, topoisomerases IIA not only simplify DNA topology [4], [17], [18] but efficiently discriminate between knots of opposite signs [19]. Importantly, DNA gyrase, topoisomerase IV and human topoisomerase IIα act preferentially on (+)sc DNA [20]–[24]. Several hypotheses have been proposed to explain these observations including DNA kinking [25], kinetic proofreading [26], three segment binding [27] and hooked juxtapositions [28]. However, recent experimental studies have firmly established that topoisomerases discern the global topology of DNA on the basis of the local geometry of DNA crossovers [21], [22], [29], [30]. The link between the crossover geometry and the sign of DNA supercoiling may represent the key to understanding these questions. However, little is known about the interplay between the DNA topology and DNA-DNA interactions.

Most current models of supercoiled DNA commonly ignore the chiral nature or sequence-dependent pattern of B-DNA which is expected to have a great impact on the geometry of DNA crossovers. Elastic rods with standard repulsive self-contact energies [31], [32] would result in left and right-handed crossover structures, encountered in (−)sc and (+)sc DNA, respectively, that are equivalent from the geometric and energetic point of view. However, it is well known that the chirality of a helix may influence the geometry of its higher order structure [33]. For example, soon after the discovery of the structure of the α-helix in proteins, the role of chirality in its hierarchic assembly into helical bundles has been postulated [34]. The model of “knob into holes” predicted that simple geometric constraints are responsible for the left-handed helical organisation of coiled coils. Probably due to the molecular dogma of electrostatic repulsion, a similar hierarchic transfer of chirality has not been envisaged for the close approach of DNA double helices. Nevertheless, crystallographic studies have clearly shown that the geometry of the B-DNA double helix can direct its supramolecular assembly through a similar “knob into hole” groove-backbone interaction [35], [36].

In the present paper, we demonstrate that due to the intrinsic helical chirality of DNA, the global topological state of DNA is asymmetrically encoded in the geometry and stability of DNA crossovers. We propose that this differential stability of crossovers may be exploited for sensing the global topology of DNA from local interactions. As an important consequence of the distinct tertiary contacts between DNA segments, supercoiled DNA of opposite signs must display drastically different physical properties. In showing that the chiral nature of the B-DNA helix profoundly affects the physical properties of the superhelices of opposite signs, our study also provides new clues that may contribute to understand the early evolutionary choices for a particular DNA topology in the cellular environment.

## Results and Discussion

### Interplay of Global Topology and Local Interactions in DNA

**Figure 1:**
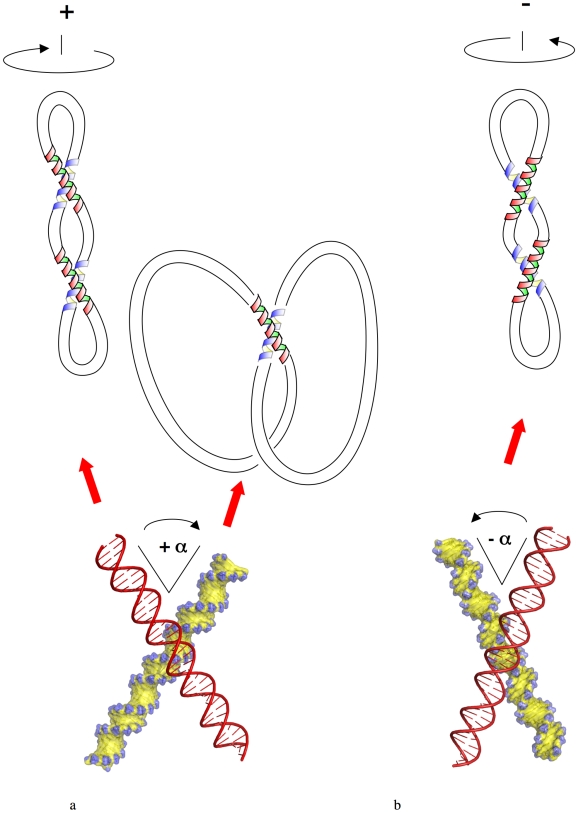
Properties of DNA crossovers and their link to DNA topology. In (+)sc DNA, the plectoneme forms a left-handed superhelix with right-handed crossovers (clockwise rotation for closing the small angle). In (−)sc DNA, the plectoneme forms a right-handed superhelix with left-handed crossovers. (a) Schematic representation of (+)sc DNA and catenanes with right-handed crossovers formed by groove-backbone interaction between two B-DNA duplexes, as found in crystallographic structures. (b) Schematic representation of (−)sc DNA with left-handed crossovers formed by the juxtaposition of the major grooves of two B-DNA duplexes, as found in crystallographic structures.

Both experimental and theoretical studies converge towards a unified picture of supercoiled DNA: an interwound plectonemic molecule whose properties are greatly influenced by ionic conditions [31], [32]. In the presence of divalent cations, closely packed regions with tight intersegmental contacts are observed [37], [38]. Moreover, the importance of such contacts for biological processes has been noted in early theoretical studies [39]. Although thermal fluctuations generate variations in the parameters of the supercoiled DNA molecules [40], independent approaches have shown that the relative orientations of the juxtaposed sites in the interwound superhelix have a narrow distribution with an absolute value for the crossover angle (α) close to 60° (see Fig. 1 for definitions) [31], [37], [41], [42]. Monte Carlo studies have shown that this orientational preference is observed even at low supercoiling densities (σ = −0.01) [42]. Importantly, several studies indicate that the crossing angles are similar in magnitude but opposite in handedness in (+) and (−) supercoiled DNA molecules [21], [22], [42]. Single-molecule manipulation studies have quantified the angle values of DNA crossovers in L-braids ((+)sc) and R-braids ((−)sc) to be around 60° and −60°, respectively [21], [22]. However, since typical models of supercoiled DNA consist of flexible tubes with repulsive self-contact energies [31], [32], the energetic difference between left-handed and right-handed DNA crossings is essentially zero.

**Figure 2:**
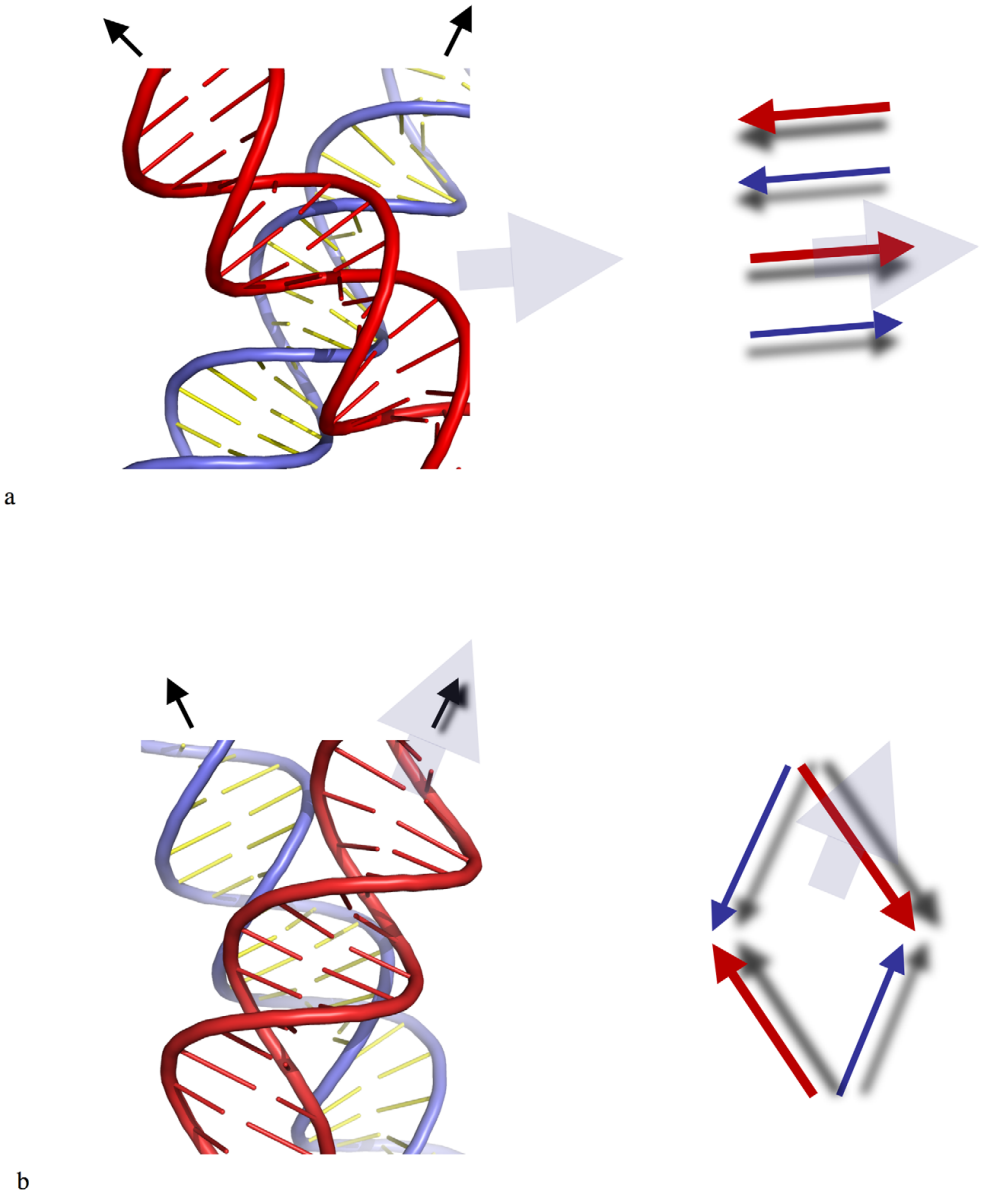
DNA chirality and geometry of crossovers. (a) Detailed view of the major groove-backbone interaction. (b) Detailed view of the major-groove/major-groove interaction. The front helix is represented in red and the back helix in blue. The thin black arrows indicate the directions of the helical axes, and the large grey arrows indicate the direction of the major groove in the back helix. On the right, the red and blue arrows indicate the backbones at the interface of the crossovers in the 5′-3′ direction. In right-handed crossovers, the backbone of one helix (red) is oriented along the direction of the major groove of the other one (blue). In left-handed crossovers, the helical axis of one helix (red) is oriented along the direction of the major groove of the other one (blue).

B-DNA double helices can form tight crossovers in crystals where the electrostatic repulsion between the negatively charged backbones is minimized. We analysed the geometry of available crossover structures crystallised in different space groups to gain insight into the organisational principles that drive DNA-DNA interaction. We classified the crossover structures according to the mode of interaction between the duplexes and determined their acute crossing angle (Table 1). In right-handed crossovers that are characterized by positive values of the crossing angle, the double helices can be mutually self-fitted by groove-backbone interaction. The backbone of one helix is inserted into the major groove of the other one (Fig. 2a). Consequently, the phosphate group can penetrate the major groove to form hydrogen bonds to the amino groups of the anchoring cytosines [35], [36], [43], [44]. The values of the crossing angle depend on the actual DNA sequence, duplex geometry and crystal packing (Table 1). Notably, most of the right-handed crosses examined to date are assembled by the major groove-backbone interaction. This interaction is sequence-dependent and mediated by divalent cations. Interestingly, this mode of assembly has also been observed in the crystal packing of nucleosome core particles (NCP) in the presence of Mn^2+^ [45] (Table 1). These self-fitted compact structures are isostructural to the stacked Holliday junction [46]. Although less frequent, minor groove-backbone interaction has also been found in crystal structures of B-DNA duplexes (Table 1). Sequence-specific interactions between the amino-groups of guanines and the phosphate groups may stabilise this type of duplex assembly. Thus, both major- and minor groove-backbone interactions appear to require GC base pairs for their sequence-dependent interactions in right-handed crossovers. Cytosine therefore constitutes a major determinant for the assembly of right-handed crossovers. This observation has previously been exploited in designing crystal anchoring points and is now widely used for diverse DNA sequences (Table 1) [35], [36], [43], [44]. Left-handed crossovers that are characterized by negative values of the crossing angles prevent the self-fitting of the double helices. As a result, B-DNA helices are juxtaposed by groove-groove interactions to minimize their electrostatic repulsion [36], [47] (Fig. 2b). This mode of interaction does not involve sequence-specific contacts between DNA segments or specific divalent cation bridges.

**Table 1:** Right- and left-handed crossovers in crystal structures of B-DNA duplexes and nucleosome core particles.

Mode of interaction	Space group	DNA sequence	cation	Crossing angle (°)	NDB ID
**Right-handed crossovers**					
Major-groove/backbone					
	R3	AC**C**GG**C**GCCACA	Mg^2+^	79	BD0022
		AC**C**GC**C**GGCGCC	Mg^2+^	77	BDL035
		AC**C**GC**mC**GGCGCC	Mg^2+^	75	BDLB83
		AC**C**GA**C**GTCGGT	Mg^2+^	73	BD0001
	R3	**CC**GC**C**GGCGG	Mg^2+^	78	BD0015
		**CC**GC**C**GGCGG	Ca^2+^	82	BD0081
		**CC**GT**C**GACGG	Ca^2+^	79	BD0080
		**CC**GG**C**GCCGG		78	BDJ039
	C121	**C**TCTCGAGAG	Ca^2+^	42	BDJ060
		**C**TTTTCTTTG		53	BDJ081
		C***C***GCTAGCGG		50	BD0028
		C***C***TCTAGAGG		46	BD0076
		C***C***AGTACTGG (Imidazole-pyrrole-hydroxypyrrole polyamide)		51	BDD002
		CCII**C**ICCII (netropsin)		38	DD0024
	P3_2_21	**C**CAACITTGG	Mg^2+^	58	BDJB43
		**C**GATCGmATCG	Mg^2+^	63	BDJB48
		**C**CATTAATGG		64	BDJ055
		**C**CACTAGTGG		62	BDJ061
	P3_1_21	GCAAA**C**GTTTGC		61	BD0047
	P4_3_	AC***C***GGTACCGGT		90	BD0003
	P1	AC***C***GAATTCGGT		73	BD0052
		ACCGA**C**GT**C**GGT		62	BD0002
		GCAGA***C***GTCTGC	Co(NH_3_)_6_^3+^	63	BD0090
	P6_1_22	CCAGTA**C**TGG	Na^+^	62	UD0029
		CC(1AP)GTA**C**TGG	Ca^2+^, Na^+^	61	BD0068
	P4_1_22	I***C***ITACIC (distamycin)	Mg^2+^	90	GDLB51
		I***C***ATATIC (distamycin)	Mg^2+^	90	GDLB50
		I***C***ICICIC (distamycin)	Mg^2+^	90	GDHB25
	P2_1_2_1_2_1_	NCP (*X. laevis*)	Mn^2+^	67	PD0287
		NCP (human)		60	PD0676
	I222	tetranucleosome		68	PD0639
Minor-groove/backbone					
	C121	CGCAATTGCG		38	BDJ069
**Left-handed crossovers**					
Groove juxtaposition Major <> Major					
	P3_1_	CCAGATCTGG Hydroxypyrrole-imidazole-pyrrole-polyamide		−60	DD0020
		CCIIICCCGG		−60	BDJB77
	C121	CTCTCGAGAG		−42	BDJ060
		CCGCTAGCGG		−50	BD0028
		CCTCTAGAGG		−46	BD0076
Minor <> Minor					
	P3_1_	CCGAGCTCGG		−60	BD0084
Major <> Minor					
	R3	CCGCCGGCGG		−78	BD0015
		CCGTCGACGG		−79	BD0080
		CCGGCGCCGG		−77	BDJ039

The cytosine bases that form hydrogen bonds with the penetrating phosphate are shown with bold characters. The cytosine bases that are located between 3.5 and 4.5 Å of the inserted phosphate are shown in bold italic.

**Figure 3:**
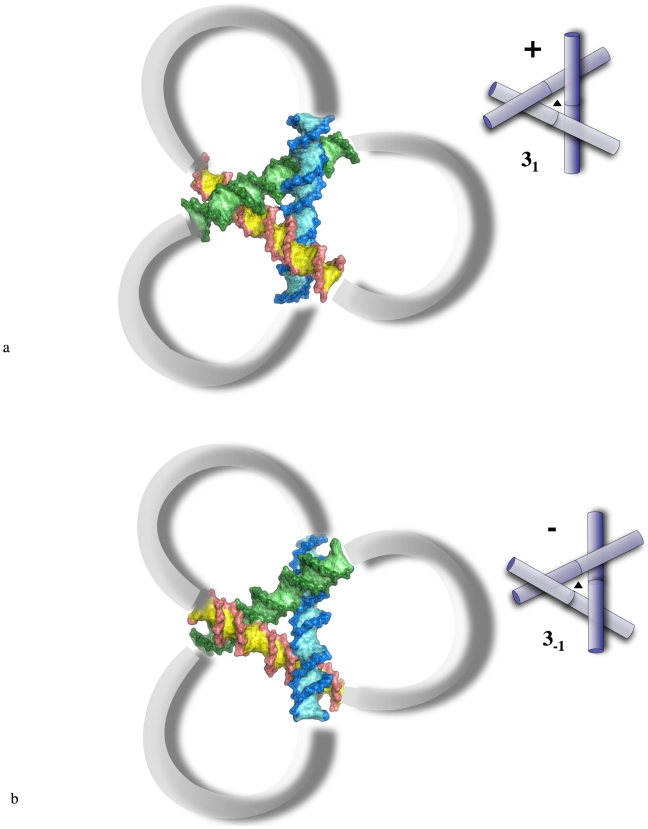
Influence of the helical chirality on the formation of DNA trefoil knots of opposite signs. Motifs produced by the combination of crossovers in the crystal structure of the decamer d(CCGCCGGCGG) (NDB id: BD0015). The loops that connect the arms are for illustration purposes only. The two trefoils alternate along a common 3-fold axis of the unit cell. (a) (+) trefoil with groove-backbone intersegmental interaction (3_1_). (b) (−) trefoil assembled with groove-groove intersegmental interactions (3_−1_). Positive trefoils adopt a significantly more compact structure than negative trefoils.

Crystal packing of DNA crossovers has also provided structural examples of more complex chiral motifs such as the heart of trefoil knots of opposite signs (Fig. 3) [36]. These structures also support the notion of hierarchical assembly of large DNA where the intrinsic helical chirality of DNA controls the distinct local geometry of DNA crossing. For steric reasons, these 3-fold symmetrical motifs correspond to the most compact structures of overlapped double helices that can be physically obtained, and in this respect, they may represent a molecular example of the heart of a so called “ideal trefoil knot” [48], [49]. However, Figure 3 clearly shows that the geometries of trefoil knots of opposite chiralities are not equivalent. The most compact structure is formed by the positive one (right-handed) that corresponds to the 3_1_ trefoil knot in which the DNA segments are self-fitted by groove-backbone interactions.

**Figure 4:**
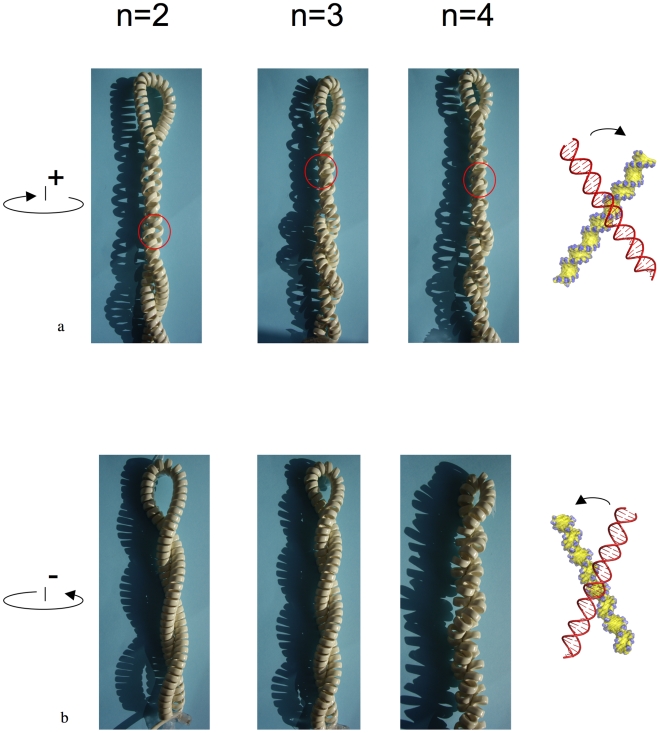
Interplay of chirality at different scales. Different inter-segmental interactions occur in superhelices of opposite signs in a simple right-handed helical telephone cord. (a) When the apical loop is turned clockwise around the longitudinal axis of the superhelix, a left-handed plectonemic superhelix equivalent to (+)sc DNA is generated. The interlocked inter-segmental contacts are indicated by red circles. These self-fitted right-handed crossovers mimic the groove-backbone interaction in DNA. Remarkably, this (+)sc plectoneme forms a stable structure that remains tightly interlocked, even after the removal of the superhelical tension. (b) When the apical loop is turned anticlockwise around the longitudinal axis, a right-handed superhelix equivalent to (−)sc DNA is generated with left-handed crossovers. Stable interlocked contacts do not appear even at a very high superhelical density. In contrast to (+)sc, the (−)sc plectoneme relaxes immediately after releasing the superhelical stress. Note that the telephone cord is slightly pulled while rotated to allow the opening of the grooves that would otherwise remain closed due to the elastic behaviour of the cord.

In a previous study, we have determined the free energy of interaction of DNA duplexes in right and left-handed crossovers as a function of divalent cation concentration in solution using molecular dynamics simulations [50]. A short-range attraction of about −4 kcal mol^−1^ between the duplexes in a right-handed crossover arrangement was observed in the presence of divalent cations [50]. This finding fits well with recent theoretical and experimental observations of close DNA-DNA interactions in the presence of divalent cations [51]–[53]. We have shown that stabilisation of DNA helices at short-range is maintained by specific major groove-backbone interactions and bridging divalent cations. In contrast, left-handed crossovers are unstable at similar ionic conditions and resulted in a swift dissociation of the helices. Without specific intermolecular interaction, DNA double helices juxtaposed by major groove-major groove interaction are stable only in the crystallographic environment but appeared to be unstable in solution. Thus the fundamental premise of energetic degeneracy of chiral crossovers in solution is not valid. Due to the helical chirality of DNA, not only the geometries but also the stabilities of crossovers of opposite handedness are inevitably different. Consequently, tertiary inter-segmental contacts in supercoiled DNA of opposite signs will also have different geometries and stabilities (Fig. 1). The left-handed superhelix formed in (+)sc DNA (overwound) favours stable right-handed crossovers self-fitted by groove-backbone interactions. In contrast, unstable left-handed crossovers juxtaposed by groove-groove interactions are formed in the right-handed superhelix of (−)sc DNA (underwound). We propose that the differential stability of tertiary contacts in supercoiled DNA of opposite signs leads to markedly different physical properties of the superhelix. The interplay of chirality at different scales can be illustrated by the behaviour of a right-handed telephone cord (Fig. 4). It is clear that the (+)sc and (−)sc cords have distinct inter-segmental interactions. At high positive supercoiling, the segments are tightly inter-digitated that resembles groove-backbone interactions in a right-handed crossover. In contrast, negative supercoiling generates a superhelix with crossover geometry similar to the groove-groove interaction in a left-handed crossover. Remarkably, the (+)sc plectoneme forms a stable structure that remains tightly interlocked even after the removal of the superhelical tension, while the (−)sc plectoneme relaxes immediately after releasing the superhelical stress. This qualitative model indicates that the chirality of a helix has a profound influence on the geometry of its superhelix that cannot be omitted in modelling supercoiled DNA, chiral DNA knots or higher-order DNA structures. To our surprise, these simple but essential geometric and energetic considerations have not been taken into account previously for modelling DNA topology.

Our hierarchical model of DNA provides new molecular insights that may contribute to the understanding of previously unexplained experimental results that show an asymmetrical behaviour of supercoiled DNA of opposite signs. For example, single DNA manipulation experiments have shown a hysteresis in the extension length when (+)sc DNA is pulled and subsequently released in the presence of divalent cations [54]. The authors attributed this observation to an electrostatic collapse of the (+)sc molecule. Clearly, additional energy would be required to disrupt stable right-handed crossovers in an interlocked structure and relax it from the stretched state. In addition, the finding that relaxed pBR322 DNA forms positive supercoils in the presence of divalent cations [55] provides further experimental support of our results. Indeed, since Mg^2+^ and other divalent cations specifically promote the formation of stable right-handed crossovers, they should preferentially condense a DNA molecule into a left-handed superhelix of (+)sc (Fig. 1a).

### Biological Implications

#### Local discrimination of different topological states of DNA

**Figure 5:**
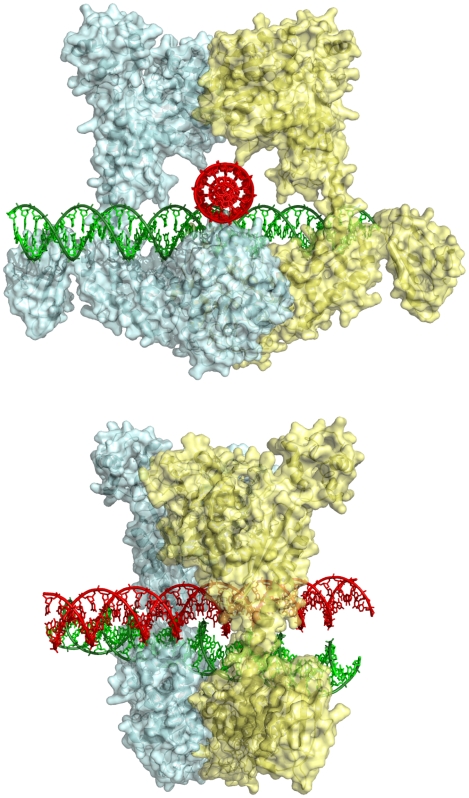
Recognition of stable right-handed crossovers by topoisomerases IIB. Model of archael topoisomerase VI of *Methanosarcina mazei* [56] clamping a right-handed crossover (top: front view and bottom: side view). In the modelling study, right-handed and left-handed DNA crosses have been docked as rigid body into the clamp delimited by the dimeric enzyme (pale cyan and yellow surfaces). One of the arms of the cross has been considered as the G-segment (gate) (green) and has been inserted into the G-segment binding groove [56], [57]. The orientation of the T-segment (transported) (red) is therefore imposed by the chirality of the crossover. The T-segment of the right-handed cross fits perfectly into the central cavity. In contrast, fitting left-handed crossovers produced steric clashes between the T-segment and the B subunit and transducer domain of the enzyme (not shown).

The effect of differential crossover geometry and stability on global DNA topology has important biological consequences. First, it provides a simple mechanism for the local discrimination of different topological states of DNA. Due to their stability in solution, right-handed DNA crossovers constitute the most probable structure of site juxtaposition at physiological conditions. Thus, right-handed crosses that occur preferentially in (+)sc DNA for geometrical reasons, should also be preferentially formed in the absence of superhelical stress, as in relaxed DNA [55], catenanes or loose knots. In contrast, the formation of unstable left-handed DNA crosses strictly require (−) supercoiling which is the normal topological state of mesophilic cells. Consequently, maintaining a constant level of (−) supercoiling prevents the formation of right-handed crosses. In other words, stable right-handed crossovers constitute the local signature of unusual topological states of cellular DNA. Indeed, positive supercoiling occurs only transiently in mesophilic cells, during replication or transcription [5]–[9]. Sensing the differential stability and geometry of DNA crossovers would be the secret of Maxwell's topological demons [17]? It is likely that topoisomerases II have evolved to clamp stable DNA juxtapositions. An interesting hypothesis is that these enzymes may also have exploited the electrostatic properties of crossovers for their catalytic mechanism of the strand-passage reaction. Type II topoisomerases catalyse the ATP-dependent transport of one intact DNA double helix, the transported segment (“T-segment”), through the gate segment that contains the enzyme-mediated transient DNA gate (“G-segment) [1]. Many biochemical studies support the view that the juxtaposed G- and T-segments bind at the interface of the B′/A′ DNA binding and cleavage core and the ATPase domains [1]. Clamping both the G- and T-segments should be greatly facilitated if there is an attractive interaction between the duplexes (“pull”). Right-handed crosses are therefore optimal candidates as substrates of the reaction (Fig. 5). In contrast, expelling the T-segment from the enzyme would be facilitated by the repulsive interaction between the DNA segments within a left-handed crossover generated by the reaction (“push”). Unstable left-handed crossovers are therefore better candidates for being the product of the strand passage reaction. Two recent structures of the topoisomerases IIB family, the archaeal topoisomerases VI, have indicated that DNA crossovers may be tightly confined into a central cavity formed within the ATPase domain and the B′/A′ domain [56], [57]. The overall organisation of these two domains is considered to be representative of all type II enzymes [56], [57]. Modelling complexes of topoisomerase VI with right- or left-handed crossovers clearly indicates that a right-handed crossover fits perfectly into the central cavity (Fig. 5). In contrast, attempts to fit left-handed crossovers produced steric clashes with the protein domains. The model represented in Figure 5 provides further support to the idea that topoisomerases II have evolved to recognise stable right-handed DNA and may also help to understand how two DNA segments can simultaneously be confined into a tight protein clamp. Indeed, crossover recognition by topoisomerases II has been a highly controversial subject because of the electrostatic repulsion between the DNA segments. Our hypothesis fits well with the observation that many type II topoisomerases act preferentially on (+)sc DNA [20]–[24] that occur transiently in front of the replication fork [8]. It is likely that the formation of sticky intersegmental interactions in (+)sc contribute to impede the progression of the replication fork (see evolutionary aspects below). This would explain, among other factors, why (+)sc might be quickly removed by type II topoisomerases [8]. Moreover, recent single-molecule measurements of the relaxation of (+)sc and (−)sc DNA by topoisomerase IV has concluded that the enzyme is highly processive on (+)sc DNA and distributive on (−)sc DNA [58]. It can therefore be speculated that the processivity of topo IV may be influenced by the stability of the crossovers.

#### Evolutionary choice of DNA topology

From an evolutionary point of view, the asymmetrical behaviour of supercoiled DNA of opposite signs may have exerted some physical constraints and contributed to orient early choices for DNA topology in the nascent DNA world. DNA topology has been the subject of adaptive pressure in organisms that live at different temperatures for maintaining the balance between the melting potential and functional stability [4], [10], [11]. For example, it is commonly thought that the underwound DNA in (−)sc facilitates the strand separation required for transcription or DNA recombination in mesophilic bacteria [3], [31], [32]. Negative supercoiling also plays an important role in linking the overall metabolic state of the cell to gene regulation [3], [4]. On the other hand, reverse gyrase is found in all hyperthermophilic archaea and bacteria [10], [11] and in some thermophilic bacteria [59]. Since this enzyme has the unique ability to introduce (+) supercoils into the DNA molecule, DNA overwinding has been thought to compensate for the destabilizing effect of high temperature. However, the picture appears to be more complex. Indeed, while plasmids of hyperthermophilic archaea that contain uniquely reverse gyrase are either relaxed or slightly positively supercoiled [60], those of hyperthermophilic bacteria [61] and some archaea [62] that have both a gyrase and a reverse gyrase are highly negative supercoiled. Although never tested, this is probably representative of chromosome supercoiling in these organisms. Thus, relaxed or (+)sc DNA appears not to be strictly required for an adaptation to high temperatures, in good agreement with studies that showed that (−)sc DNA can be stable at high temperature and that (+) supercoiling does not increase the thermal stability of closed circular DNA [63]. However, reverse gyrase that is a distinctive trait for adaptation to high temperatures may play an alternative role, for example, in protecting DNA against thermal degradation [64]. These studies show therefore that slightly overwound or relaxed DNA is not indispensable for life at high temperature, and conversely that the presence of underwound DNA is not detrimental for hyperthermophilic organisms.

In contrast to life at high temperature that can tolerate various topological states of DNA, adaptation to mesophilic life constrains much more the topology of DNA. Indeed, the genome of mesophilic organisms, including bacteria, archaea and eukarya, is (−) supercoiled. All mesophilic bacteria have a DNA gyrase that introduce (−) supercoiling in a plectonemic form [4]. Particularly interesting is the case of mesophilic archaea. They have either acquired a gyrase that introduce negative supercoiling, or histones that wrap DNA into toroidal supercoils [4], [10]. In other words, mesophilic organisms appear to have evolved to strictly avoid the presence of permanently relaxed or (+)sc DNA in their genome. Our study brings another piece of information to this complex puzzle by providing new insights about the intrinsic properties of the B-DNA double helix. It is likely that, among other physical properties of DNA, such as its anisotropic flexibility [65], [66], or the fact that DNA is more easily untwisted than overtwisted, the differential stability of chiral crossovers has influenced the choice of DNA topology in mesophilic cells.

In particular, the formation of stable right-handed crossovers in relaxed or (+)sc DNA may have posed challenges to mesophilic cells. Indeed, from a functional point of view, right-handed DNA crosses can be viewed as a Janus-like DNA structure. The stable and specific self-assembly of double helices can be useful for closely packaging DNA into higher-order DNA structures. However, right-handed crossovers may have a detrimental effect by impeding the global dynamics of the genome, if they occur without control within a plectonemic supercoiled DNA. Therefore, these two opposite features may have lead to different evolutionary strategies to adapt to mesophilic conditions where weak interactions that occur within right-handed crossovers can be expected to be stable.

First, in gyrase-containg bacteria and archaea, the dynamics of plectonemic DNA supercoiling plays an important role in promoting interactions between remote sites in processes such as transcription initiation and site-specific recombination [2]–[4]. However, several studies have shown that some particular local inter-segmental contacts may impede the dynamics of supercoiled DNA and affect functions [12]–[14]. Similarly, divalent cations that promote formation of stacked 4-way junctions [67] considerably slow down the kinetics of spontaneous branch migration [68]. Our study therefore predicts that in the presence of divalent cations, the stable inter-segmental interactions should make (+)sc DNA significantly more “sticky” than (−)sc DNA, along GC rich sequences. Maintaining permanent (−) supercoiling could therefore be viewed as preventing sticky interactions and promoting the “fluidity” required for various functions. Our model can also account for the observation that hyperthermophilic archaea tolerate other topological states of DNA, such as the relaxed or slightly (+)sc states. Indeed, higher temperatures would decrease the stability of right-handed crossovers and restore the relative mobility of DNA segments.

Second, wrapping DNA around histones in mesophilic archaea and eukarya can be viewed as an alternative mode of adaptation to the presence of sticky DNA-DNA interactions in their genome. It can be speculated that this regular mode of DNA packaging allows the organism to precisely control the position of right-handed crosses and to exploit their physical properties. For example, it has been proposed that DNA self-fitting may contribute to stabilise the interactions between nucleosomes [35] or DNA linkers [36] within the chromatin fibre [69]. A recent all-atom model of the chromatin fibre that involves multiple DNA-DNA interactions [70] and the observation of groove-backbone interactions between nucleosomal DNA in the crystal packing of nucleosome core particles (Table 1) [45] reinforce this hypothesis.

## Materials and Methods

High-resolution crystal structures of DNA and DNA-drug complexes were studied from the Nucleic Acid Database [71]. Although the DNA structures available in the database may not represent all the possible forms of interactions between DNA helices uniformly, we focused our attention on the common structural features, but noting the subtle variations that can influence stability. Symmetry-related helices were generated with standard matrix transformations and visualised by VMD [72] and Pymol [73]. Structural analysis was carried out using the program Curves [74] and scripts developed in our laboratory. The angle of the crossover was defined as the angle between the best linear axes of the individual duplexes obtained from Curves.
